# Does the use of the Informed Healthcare Choices (IHC) primary school resources improve the ability of grade-5 children in Uganda to assess the trustworthiness of claims about the effects of treatments: protocol for a cluster-randomised trial

**DOI:** 10.1186/s13063-017-1958-8

**Published:** 2017-05-18

**Authors:** Allen Nsangi, Daniel Semakula, Andrew D. Oxman, Matthew Oxman, Sarah Rosenbaum, Astrid Austvoll-Dahlgren, Laetitia Nyirazinyoye, Margaret Kaseje, Iain Chalmers, Atle Fretheim, Nelson K. Sewankambo

**Affiliations:** 1 Makerere University, College of Health Sciences New Mulago Hospital Complex, PO Box 7072, Kampala, Uganda; 2 Norwegian Institute of Public Health, PO Box 4404, Nydalen, N-0403 Oslo Norway; 3 University of Oslo, Postboks 1130, Blindern, 0318 Oslo Norway; 4 University of Rwanda, 101, KK 19 Av., University Avenue, PO Box 5229, Kigali, Rwanda; 5 Great Lakes University of Kisumu, PO Box 2224-40100, Kisumu, Kenya; 6 James Lind Initiative, Summertown Pavilion, Middle Way, Oxford, OX2 7LG UK

**Keywords:** Critical thinking, Critical appraisal, Higher-order thinking, Meta-cognition, Treatment claims, Health literacy, Evidence-based health care, EBM teaching resources, Primary school curriculum, Science teaching

## Abstract

**Background:**

The ability to appraise claims about the benefits and harms of treatments is crucial for informed health care decision-making. This research aims to enable children in East African primary schools (the clusters) to acquire and retain skills that can help them make informed health care choices by improving their ability to obtain, process and understand health information. The trial will evaluate (at the individual participant level) whether specially designed learning resources can teach children some of the key concepts relevant to appraising claims about the benefits and harms of health care interventions (treatments).

**Methods:**

This is a two-arm, cluster-randomised trial with stratified random allocation. We will recruit 120 primary schools (the clusters) between April and May 2016 in the central region of Uganda. We will stratify participating schools by geographical setting (rural, semi-urban, or urban) and ownership (public or private).

The Informed Healthcare Choices (IHC) primary school resources consist of a textbook and a teachers’ guide. Each of the students in the intervention arm will receive a textbook and attend nine lessons delivered by their teachers during a school term, with each lesson lasting 80 min. The lessons cover 12 key concepts that are relevant to assessing claims about treatments and making informed health care choices. The second arm will carry on with the current primary school curriculum.

We have designed the Claim Evaluation Tools to measure people’s ability to apply key concepts related to assessing claims about the effects of treatments and making informed health care choices. The Claim Evaluation Tools use multiple choice questions addressing each of the 12 concepts covered by the IHC school resources. Using the Claim Evaluation Tools we will measure two primary outcomes: (1) the proportion of children who ‘pass’, based on an absolute standard and (2) their average scores.

**Discussion:**

As far as we are aware this is the first randomised trial to assess whether key concepts needed to judge claims about the effects of treatment can be taught to primary school children. Whatever the results, they will be relevant to learning how to promote critical thinking about treatment claims.

Trial status: the recruitment of study participants was ongoing at the time of manuscript submission.

**Trial registration:**

Pan African Clinical Trial Registry, trial identifier: PACTR201606001679337. Registered on 13 June 2016.

**Electronic supplementary material:**

The online version of this article (doi:10.1186/s13063-017-1958-8) contains supplementary material, which is available to authorized users.

## Background

Health literacy, as defined by Healthy People 2010, is ‘the degree to which individuals have the capacity to obtain, process and understand basic health information needed to make appropriate health care decisions’ [1]. There has been an explosion in communication avenues for all types of information, including health, and children as well as adults are bombarded with all sorts of claims about the benefits and harms of treatments. This includes claims about conventional medicines, herbal medicines and nutritional therapies, dietary supplements, cleansing therapies, massage, reflexology and many other types of treatments. Belief in false claims about treatments causes harm and wastes resources. Not believing reliable claims means that effective treatments are not used. These problems are especially serious in resource-poor settings, where people have few resources to waste and a large burden of disease.

Several studies have concluded that people’s ability to assess health information is generally low and, in most cases, lacking [2–9]. While some studies have assessed adult health literacy and parental health literacy, only a few have focussed on health literacy among children and all these studies have been done in high-income countries [10–13]. Health-related knowledge, attitudes and behaviours developed during childhood are increasingly being recognised as foundational, deeply rooted and resistant to change later, when children become adults [14, 15], yet we have not been able to identify any studies that have addressed children’s assessment of claims about treatment effects. Children between the ages of 10 and 12 years in some countries are taught about fair tests and critical appraisal [16], but not with a focus on health or specifically teaching them to assess claims about the effects of health care interventions (which we will refer to as treatments).

An overview of six systematic reviews of educational interventions in low- and middle-income countries found 227 studies in total that reported student learning results [17]. None of these studies addressed health or scientific literacy or critical thinking more broadly. A systematic review of the effects of instruction on the development and enhancement of critical thinking skills at any age, and in any setting, found 49 studies of the effects of strategies for teaching primary school children (aged 6 to 10 years) to think critically [18], none of which focussed specifically on health literacy or assessing claims about treatment effects. Similarly, reviews that have focussed specifically on teaching children critical appraisal skills in relation to health have not found any studies that evaluate the effects of strategies to teach these skills to primary school children anywhere [19–21].

Teaching children how to assess claims about the effects of treatments might be effective for several reasons. First, children are capable of learning about fair tests and critical appraisal between the ages of 10 and 12 years and teaching these basic skills is already part of the curricula in some countries [16].

Second, it is possible to reach a large segment of the population before they drop out of school, since large numbers of children drop out after primary level in low-income countries [22–24]. The UN Educational, Scientific and Cultural Organisation (UNESCO) has estimated that 68% of children in Uganda who enrol in primary school are likely to drop out before finishing the prescribed 7 years [25]. Primary school in Uganda comprises seven classes from primary one (grade 1) to primary seven (grade 7) completed during a period of 7 years, with the official age range for primary education level being 6–12 years [26]. However, children attending primary school are generally aged between 6 and 17 years or even older in some schools, especially in conflict-torn areas [24].

Third, teaching children at primary school level to assess claims about treatments can capitalise on children’s natural curiosity and enthusiasm to learn.

Fourth, there are opportunities for children to share what they have learned with other children and family members (parents or guardians). In addition, primary schools play an important role in many communities in sub-Saharan Africa, particularly Uganda, where 49% of the population are below the age of 15 years [27]. Teaching basic concepts in schools about how to assess claims about the effects of treatments might create opportunities for both the children and their families to learn the critical appraisal skills that they need when assessing the benefits and harms of treatments. Finally, a good foundation for a healthier society might result from teaching children to ask questions about treatment claims and how to assess health information about treatment effects before the formation of problematic health attitudes and behaviours in adulthood [14, 15].

For these reasons, we have developed and pilot-tested resources to help teach children how to assess claims about the benefits and harms of treatments. We will evaluate the effects of these resources on knowledge in a randomised comparison with the standard curriculum.

In parallel, we have developed a podcast to teach some of the same concepts to parents of these children. We will test the effects of the podcast in a linked individual-randomised trial [28]. In that trial, we will randomly allocate 500 parents of some of the children in this trial to either listen to the podcast or to typical public service announcements about health issues. The parents will be volunteers, recruited from both intervention and control schools. We will use the same outcome measure in both trials, and we will measure the effects of the podcast on the children of parents included in that trial, and the effects of the primary school resources on those parents.

## Objectives

This research will address the following question: Does use of the Informed Healthcare Choices (IHC) school resources improve children’s ability to assess claims about treatment effects?

The primary objective of this study is to measure the impact of the IHC school resources on children’s ability to assess claims about treatment effects.

Secondary objectives of the study are to measure effects on the proportion of children who achieve a score indicating mastery of the 12 concepts covered by the primary school resources, their understanding of and ability to apply specific concepts relevant to the assessment of claims about treatment effects, intended behaviours, self-efficacy, attitudes towards school, attendance and academic achievement.

## Methods

**Fig. 1:**
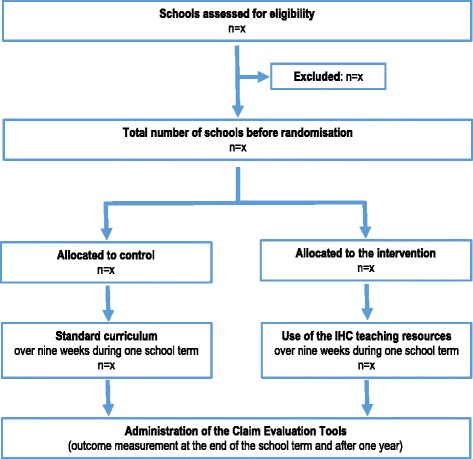
Study flow chart

This will be a two-arm, cluster-randomised trial as illustrated in the flow chart below (Fig. 1). Because the intervention will be delivered by teachers in primary schools, we will randomise schools rather than individual children. However, the objective – improving children’s ability to assess claims about treatment effects – pertains to individual children.

## Study population and random allocation

The study population will consist of primary-five (grade-5) children enrolled in school at the time of the study. Primary-five children in Uganda are, on average, between 10 and 12 years old, but in some cases older, especially in conflict prone areas. Within each participating school, we will include all primary-five children.

We will ask the head teachers of the participating schools to select the primary-five teachers who will deliver the lessons using the IHC primary school resources (or who would have done so in the control schools). There will be no inclusion or exclusion criteria for the teachers, other than that they provide written consent to participate in the trial. The recruitment process will be done starting 1 April 2016 to 31 July 2016.

### Inclusion and exclusion criteria

#### Inclusion

Public and private primary schools in the central region of Uganda will be eligible to participate. Primary-five students in those schools will be included in the study.

#### Exclusion

We will exclude international schools, special needs children’s schools for the deaf and blind, and schools that participated in the user testing and piloting of the resources. Children in primary level classes other than primary five will be excluded from participating in the study. For practical reasons, we will exclude schools that are too difficult to access.

Only schools which agree to participate and sign a Consent Form for participation will be included in the trial.

### Sampling technique

We will use a multistage sampling technique in which we will first draw a sample of districts from all the districts in the central region in Uganda. In the second stage we will randomly sample schools proportionately from the selected districts, stratifying by school setting (urban, semiurban and rural areas), and further by ownership (privately funded and government-aided schools). According to the Uganda Bureau of Statistics, urban areas in Uganda are defined as gazetted cities; municipalities and town councils with a population of over 2000 persons; rural areas comprise villages in remote or isolated areas, usually with a population of less than 2000 people; and semi-urban areas are those found on the periphery of gazetted cities and municipalities with a population of close to 2000 people [26]. We will select a minimum of 110 schools at random using on-line software (www.sealedenvelope.com).

### Random allocation

Schools will form the basic units (clusters) for allocation. Each school will be numbered and listed, and the study arm allocation will be determined using computer-generated randomisation, with equal numbers of the schools allocated to each arm in each stratum.

A statistician who is not a member of the research team will use computer-generated allocation sequences (www.sealedenvelope.com) to randomly allocate schools to either the control or intervention arm. No changes to allocation will be made subsequent to this.

We will use a block size of 4, with equal allocation ratios in each block and strata. The lists for each district will be randomly generated as shown in the table below (Table 1).

**Table 1:** Examples of random allocation sequence

Block identifier	Block size	Sequence within block	Treatment	Location	Ownership	Code
1	4	1	Group A	Rural	Government	FZ4
1	4	2	Group A	Rural	Government	MG9
1	4	3	Group B	Rural	Government	NC5
1	4	4	Group B	Rural	Government	ID9
2	4	1	Group B	Rural	Government	VO9
2	4	2	Group B	Rural	Government	UA4
2	4	3	Group A	Rural	Government	KB5
2	4	4	Group A	Rural	Government	OQ6

As shown above (Table 1), the statistician will prepare a randomisation list with unique codes and corresponding allocation groups for each participating school. We will prepare separate lists for each stratum; i.e. rural-government for a rural school owned by government, rural-private for a rural school owned privately; suburban-private for a suburban school that is owned privately, suburban-government for a suburban school that is owned by government, urban-private for an urban school owned privately, and urban-government for an urban school that is government owned. These will contain only the participant school’s study code. Study allocation groups (intervention or control) corresponding to each study code will be inserted in envelopes and sealed. Every envelope will contain its study code as a label. For example, if the school is located in a rural area and it is a government school, a list will be prepared from the general list for that particular district. Below is an example of the study groups with corresponding study codes (Table 2).

**Table 2:** Example of study groups and corresponding study codes

Treatment	Code
Group A	FZ4
Group A	MG9
Group B	NC5

## The intervention

The IHC primary school resources include a textbook and a teachers’ guide [29, 30]. We developed the resources iteratively between 2013 and 2015, using brainstorming, pilot testing and user testing. We began by identifying 32 concepts that people need to understand and apply to be able to assess treatment claims and make informed health care choices [31] and prioritising concepts that are relevant to primary school children [32]. There are six groups of concepts (Table 3).

**Table 3:** Six groups of concepts that people need to understand and apply to be able to assess treatment claims and make informed health care choices [31]

1. Recognising the need for fair comparisons of treatments	
2. Judging whether a comparison of treatments is a fair comparison	
3. Understanding the role of chance	
4. Considering all the relevant fair comparisons	
5. Understanding the results of fair comparisons of treatments	
6. Judging whether fair comparisons of treatments are relevant	

Based on the findings of pilot testing, we reduced the number of concepts addressed in the resources to 12 concepts (Table 4), with the intention of developing additional resources in the future to introduce new concepts and reinforce understanding of those concepts. This approach is consistent with the principles of a ‘vertically aligned’ or spiral curriculum [33] which specifies where learners should begin and how they should progress. It avoids the trap of trying to teach or learn everything about a topic on the first cycle and helps to prevent learners being unprepared at later stages.

**Table 4:** Twelve key concepts that are taught in the Informed Healthcare Choices (IHC) primary school resources^a^

1. Recognising the need for fair comparisons of treatments
1.1 Treatments may be harmful
1.2 Personal experiences or anecdotes (stories) are an unreliable basis for assessing the effects of most treatments
1.4 Widely used treatments or treatments that have been used for a long time are not necessarily beneficial or safe
1.5 New, brand-named, or more expensive treatments may not be better than available alternatives
1.6 Opinions of experts or authorities do not alone provide a reliable basis for deciding on the benefits and harms of treatments
1.7 Conflicting interests may result in misleading claims about the effects of treatments
2. Judging whether a comparison of treatments is a fair comparison
2.1 Evaluating the effects of treatments requires appropriate comparisons
2.2 Apart from the treatments being compared, the comparison groups need to be similar (i.e. ‘like needs to be compared with like’)
2.5 If possible, people should not know which of the treatments being compared they are receiving
3. Understanding the role of chance
3.1 Small studies in which few outcome events occur are usually not informative and the results may be misleading
4. Considering all the relevant fair comparisons
4.1 The results of single comparisons of treatments can be misleading
5. Understanding the results of fair comparisons of treatments
5.1 Treatments usually have beneficial and harmful effects

^a^The numbers indicate the grouping of the concepts (Box 1) and the numbering of the concepts in each group [31]

**Fig. 2:**
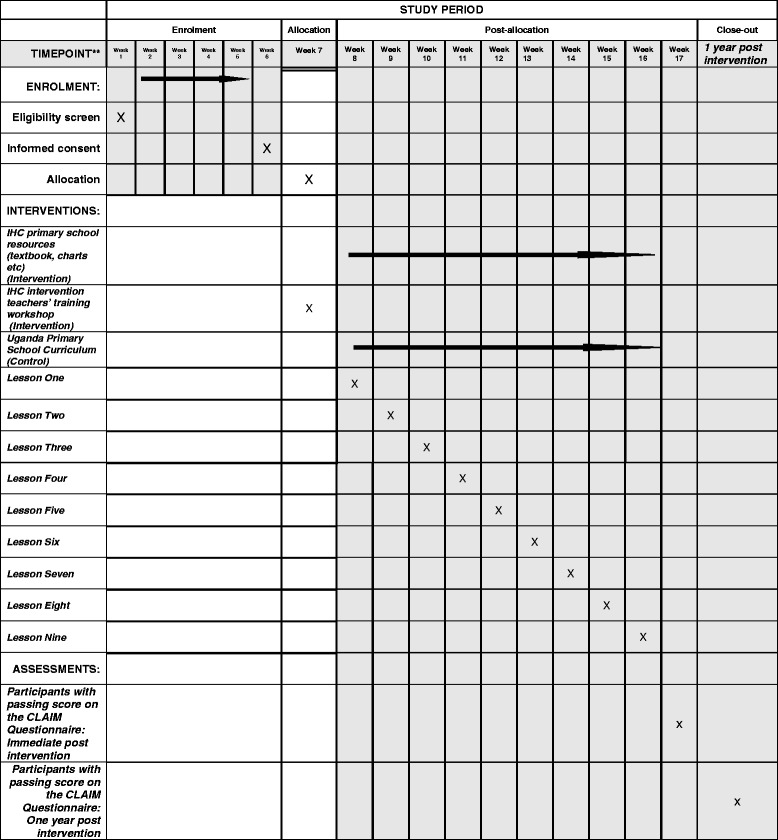
Enrolment schedule

We designed the resources to be taught over a period of 9 weeks, with one double lesson per week during a single term and 1 h for completing the Claim Evaluation Tools. There are three school terms per year in Ugandan primary schools, with school terms ranging between 12 and 14 weeks per term, and lessons are taught in 40-min periods [24]. In addition to reducing the number of concepts introduced initially, we increased the time for each lesson from one to two periods (double lessons) to address the major barrier we found in the pilot testing, which was insufficient time (see Fig. 2, which equates to the SPIRIT figure for this trial).

### The textbook, exercise books and reminders

**Fig. 3:**
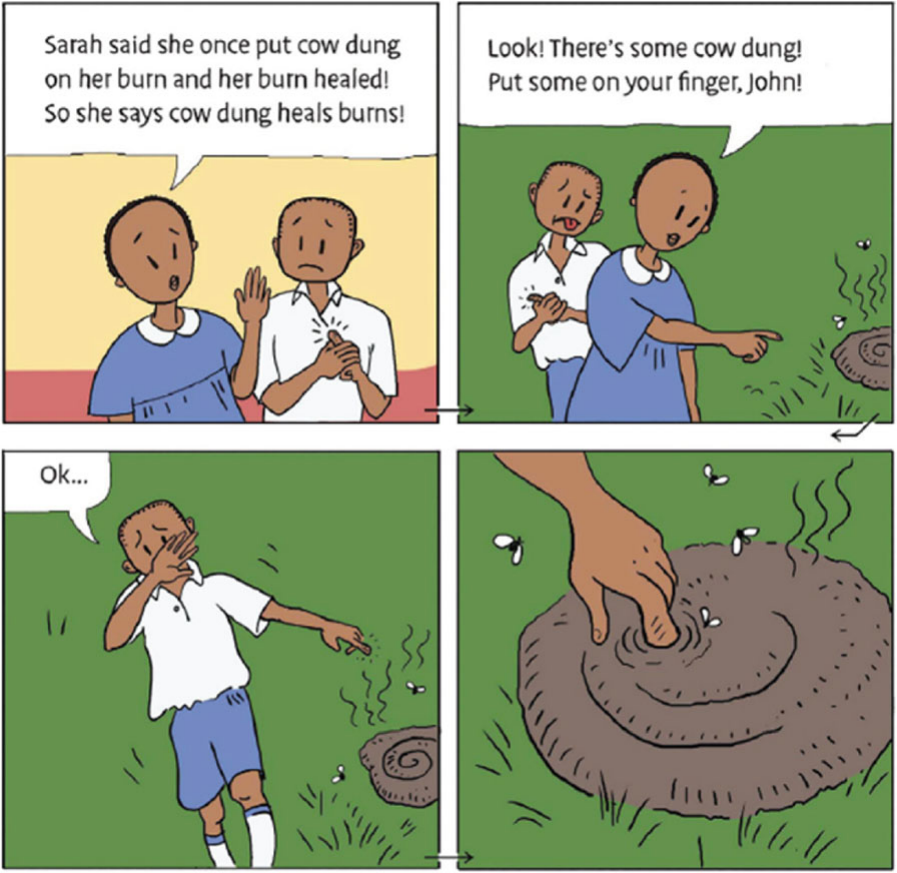
An excerpt of the comic book story

The textbook consists of a story told in a comic book format (Fig. 3), classroom activities, exercises, a checklist summarising the main lessons, and a glossary. In addition to the textbooks, we will provide each school with an exercise book for each pupil, a poster of the checklist for the classroom, and the lyrics and music to a song that includes reminders of key concepts.

The contents of the book are as shown in Table 5.

**Table 5:** Contents of the textbook

Introduction	
Chapter 1 Health, treatments and effects of treatments	
John and Julie learn about *claims* about treatments	
Chapter 2 Someone’s experience using a treatment	
Chapter 3 Other bad bases for claims about treatments (Part 1)	
Chapter 4 Other bad bases for claims about treatments (Part 2)	
John and Julie learn about *comparisons* of treatments	
Chapter 5: Comparisons of treatments	
Chapter 6: Fair comparisons of treatments	
Chapter 7: Big enough fair comparisons of treatments	
John and Julie learn about *choices* about treatments	
Chapter 8: Advantages and disadvantages of a treatment	
Review	
Chapter 9: Review of what is most important to remember from this book	

### The teachers’ guide

The teachers’ guide includes the following for each chapter, in addition to the chapter from the textbook:

- The objective of the lesson
- A lesson preparation plan
- A lesson plan
- A list of materials that the teacher and children will need
- A synopsis of the story
- Keywords in the chapter
- Review questions to ask the children after reading the story
- Extra examples for illustrating the concepts
- Background about the claims used in the story to illustrate the concepts
- Instructions for the classroom activity
- Answers and explanations for the activity
- Answers and explanations for the exercises
- Background for the teacher, explaining the concepts using language and examples that are appropriate for teachers and keyword definitions for teachers

### Delivery of the intervention

We will contact participating schools 2 to 3 months before the start of the trial and invite all participating teachers in the intervention group to attend a 2-day introductory meeting. At the meeting we will inform them about the study objectives and procedures, including the Claim Evaluation Tools that we will use as an outcome measure, introduce them to the IHC primary school resources, and address any queries or concerns that may arise. We will discuss the general nature of the evaluation tool, but will not give the teachers copies of the questionnaire. We will try to contact or visit all the teachers in the intervention group who are unable to attend a meeting.

At least 1 week before the trial commences (and prior to the introductory meeting) we will give teachers in the intervention group the teachers’ guide to enable them to familiarise themselves with the content and prepare a semester plan for delivering the lessons. We will deliver the textbooks to the schools in the intervention group at least 1 week before the trial starts. We will use class lists provided by the school heads to ensure that each school receives an adequate number of books for all the primary-five children in the school.

To help ensure that the intervention is delivered as planned, we will monitor delivery of the intervention. We will do this by following guidelines of the Ministry of Education school supervisory timetable. These allow for follow-up of newly introduced programmes within schools [34]. We will encourage the teachers to make summaries after reading each chapter in the teachers’ guide in preparation for the lesson and we will ask them to hand these in to the study team after the intervention period. This will help to ensure that the teachers read the teachers’ guide in preparation for the lessons.

We will contact the schools allocated to the control group at the beginning of the school term to inform them about the study and study procedures and the evaluation tool that we will use as an outcome measure. We will tell them that they will receive the IHC primary school resources at the end of the study, but we will not introduce them to the resources or invite them to the introductory meeting.

## Outcome assessment

We will use the Claim Evaluation Tools as the outcome measure, as applied at the level of individual children. The Claim Evaluation Tools consist of multiple choice items that assess an individual’s ability to apply 32 concepts that people must be able to understand and apply to assess treatment claims and to make informed health care choices [34]. The Claim Evaluation Tools have been developed based on extensive qualitative and quantitative feedback from methodological experts, health professionals, teachers and members of the public [34].

The questionnaires have also been subject to psychometric testing including Rasch analysis on two occasions. The first test comprised a diverse sample of over 1000 people including primary-five children exposed to pilot versions of the IHC school resources, children who were not exposed, and adults with very little or no exposure and adults who are familiar with the concepts [35]. Questions covering 24 of the 32 concepts were administered as written questionnaires in English. In this test, the items were found to have high reliability (Cronbach’s alpha = 0.81), and to be unidimensional (that is, there was no evidence of subdimensions measuring different traits). Furthermore, there was weak or no dependence among items (that is, no items were found to be redundant). Children who participated in the pilot scored better than other children, and most of the questions did not over- or under-discriminate or function differently across subgroups of participants. However, some of the questions were too difficult, particularly for children with poor reading skills.

After removing some questions, we modified the remaining questions that did not perform well according to the Rasch analysis, and revised and simplified the text where needed. The items were also translated to Luganda and adapted for audio administration. In the second psychometric testing, the Claim Evaluation Tools were administered to a similar sample as described in the first psychometric test, but approximately half of the sample received the items as a written questionnaire in English and the other half received the Luganda audio versions. The results of this test suggested that the items administered in English performed very well according to the Rasch model, and with high reliability. Furthermore, the items were also less difficult than what was found in the first psychometric testing before the revisions. The results also suggested that the Luganda versions of items had evidence of under-discrimination and differential item functioning in seven out of 29 items. These items were revised to improve fit to the Rasch model.

Based on these two psychometric tests, a selection of 24 multiple choice items addressing the 12 concepts that the IHC primary school resources cover will be used (see: Additional file 1). Each key concept is evaluated by two items. We chose items with high reliability (fit to the Rasch model) and those with an appropriate difficulty level.

The Claim Evaluation Tools also include items that assess intended behaviours, self-efficacy and attitudes associated with assessing claims and finding evidence, as well as items assessing satisfaction with the intervention relevant for primary school children (see: Additional file 1). In addition, we have included four questions that assess literacy, which we will use as a covariate in exploratory analyses, and questions about attitudes towards school. We will also compare attendance and academic achievement (using end-of-term examinations) between children in the two comparison groups.

Children in both arms of the trial will complete the questionnaire in their classrooms at the end of the term. Research assistants will ensure that the questionnaires are delivered on time, that the children are given one full hour to answer the questions, as is current practice for primary school exams in Uganda, and that the questionnaires are collected and returned to the study investigators.

### Absolute standard (for passing scores)

We will use an absolute (criterion referenced) standard to set a passing score for the version of the Claim Evaluation Tools that we will use; i.e. based on how much the children know and are able to apply. Children will be counted as ‘passing’ or ‘failing’ depending on whether they meet a specified criterion. We used a combination of Nedelsky’s and Angoff’s methods to determine the criterion [36–38], which is a cut-off for a passing score, as described in Additional file 2. In addition, we determined a second cut-off for a score that indicates mastery of the 12 concepts, using the same methods, as described in Additional file 2. The criterion for passing is a minimum of 13 out of 24 questions answered correctly. The criterion for mastery is a minimum of 20 out of 24 questions answered correctly.

To enable a sensitivity analysis, we will administer the Claim Evaluation Tools verbally to a sample of children at each school to enable estimation of the impact that literacy might have on the scores that the children achieve on the written version.

### Primary outcomes

1. The difference between the intervention and control groups in the proportion of children with a *passing* score (see above)
2. The mean difference in the score (number of correct answers) for all of the questions that assess their ability to apply the 12 concepts that are included in the IHC primary school resources

### Secondary outcomes

1. The mean difference and the difference between the intervention and control groups in the proportion of children with a passing score for a subgroup of children to whom the Claim Evaluation Tool will be administered orally
2. The difference between the intervention and control groups in the proportion of children with a score indicating *mastery* of the concepts (see above)
3. The difference between the intervention and control groups for each concept and for the questions intended to measure their understanding of the concepts
4. Differences in intended behaviours and self-efficacy (see: Additional file 1)
5. Differences in attitudes towards science and school (see: Additional file 1)
6. Differences in attendance and academic achievement as indicated by school marks

We will ask the participating schools to provide us with school attendance records and summary score sheets containing all pupils’ end-of-term examination scores. The summary score sheet contains percentage scores for each end-of-term examination, each pupil’s position in class, and a total score aggregated across subjects (Table 6). The children receive marks for English, mathematics, social studies and science. We will measure the mean difference between the intervention and control groups for each subject and for their total score (points awarded).

**Table 6:** Ranges of marks and points awarded for each subject

Exam score (out of 100)	Points awarded	Marks
80–100	1	Distinction 1
70–79	2	Distinction 2
65–69	3	Credit 3
60–64	4	Credit 4
55–59	5	Credit 5
50–54	6	Credit 6
45–49	7	Pass 7
35–44	8	Pass 8
Below 35	9	Failure

We will measure all of the above outcomes again after 1 year.

## Blinding

The comparison will not be blinded. All of the participants in the trial will be informed of the purpose of the study and will know whether they are in the intervention or control arm. The head teachers and the district education officers will be informed of the purpose of the study when they are recruited. The primary-five teachers in both arms of the trial will be informed of the purpose of the study prior to the delivery of the intervention. Children in both arms of the trial will be informed of the purpose of the Claim Evaluation Tools when they are asked to complete them. They will also be asked to put their names on the questionnaires and will be told that they and their teachers will be told their scores.

## Data collection and management

We will ask the primary-five teachers to complete a brief form at the beginning of the term to collect baseline data about the teachers in both arms of the trial (see: Additional file 3). This will include their age, gender, level of education, number of years teaching, and subjects taught. Research assistants will collect missing information by contacting the teachers by telephone or visiting the schools.

Two research assistants will independently enter data from these forms and the Claim Evaluation Tools into a database using EpiData [39]. To reduce the number of unclear or missing values, the research assistants will check all the questionnaires at the schools when they collect them and clarify any unclear entries immediately.

## Analysis

For the primary and secondary outcomes, we will use mixed models with a random effects term for the clusters and the stratification variables modelled as fixed effects, using logistic regression for dichotomous outcomes, linear regression for continuous outcomes and Poisson regression for count outcomes. For the questions that assess applied knowledge or understanding, missing values will be counted as wrong answers.

For each outcome, we will report the proportion, mean and standard deviation, or median and interquartile range for each group, the estimated difference, the estimated confidence interval for the difference, and the *p* value from the statistical model. For questions about intended behaviours and self-efficacy (see: Additional file 1), we will dichotomise the responses (e.g. ‘very unlikely or unlikely’ versus ‘very likely or likely’) in the analysis and we will report the proportions of children for each of the four response options.

### Subgroup and exploratory analyses

Based on data from a pilot study, we anticipate that many of the children will have poor reading skills. This might impede their ability to comprehend the content of the textbook and to answer questions in the Claim Evaluation Tools. We will explore whether there are differences in the effect of the intervention for children with advanced reading skills (all four literacy questions in the evaluation tool answered correctly) versus basic reading skills (both basic questions correct and one or two of the advanced reading questions wrong) versus lacking basic reading skills (one or both of the basic reading skills questions wrong). We will conduct tests for interaction for the primary outcomes, and we will use published guidelines to interpret the results of these subgroup analyses [40, 41].

Parents of 500 children in each group will be recruited to participate in a parallel trial evaluating the effects of a podcast designed to teach the parents of primary school children in areas around the participating schools, concepts that they need to understand and apply to assess treatment claims [28]. We will evaluate whether the combination of the IHC primary school resources and podcasts improves outcomes compared to the primary school resources alone by testing for interaction between IHC primary school resources and the podcast in the statistical models as described above; the main effects of the podcast will also be included in these analyses.

## Sample size

We used the University of Aberdeen Health Services Research Unit’s Cluster Sample Size Calculator [42] to calculate the sample size with the following assumptions:

- Children per cluster = 70
- Intraclass correlation coefficient (ICC) = 0.5, based on ICCs from a meta-analysis of randomised trials of school effectiveness which found ICCs to be higher than those reported for test scores in the USA [43]
- The proportion of children expected to achieve a passing score without the intervention, based on findings from pilot testing = 0%
- The smallest difference we want to be able to detect = 10%
- Alpha = 0.05
- Power = 90%

Based on these assumptions, we would need a total of 50 schools in each arm for the study to have 90% power to detect a difference of 10% between the two groups. Allowing for a loss to follow-up of up to 10% (for schools where it is not possible to administer the Claim Evaluation Tools at the end of the term), we estimate that we need a minimum of 55 schools in each group. This would provide more than 90% power to detect a mean improvement of one more correct answer for the 12 concepts covered by the primary school resources.

More specific information about the trial and the protocol is summarised in the Standard Protocol Items: Recommendations for Interventional Trials (SPIRIT) Checklist (see: Additional file 4).

## Safety monitoring and adverse events

The National Council of Science and Technology in Uganda has given this study a very low rating for risk to participants. Nonetheless, we will monitor unexpected adverse events and problems that might pose risks to the children or others by asking teachers to record these and report them to the investigators or, if relevant, to the Makerere University College of Health Sciences Institution Review Board. Teachers in the intervention arm of the trial will be given contact information at the start of the trial and instructions for recording adverse events and problems in the journals that they will be asked to keep (see: Additional file 5).

## Stakeholder involvement

A teachers’ network has contributed to the development of the primary school resources and plans for this study, and several schools have participated in piloting and user testing the resources. Participants in the study, as well as school authorities, will be fully informed of the purpose of the study. They will not have been involved in the piloting or user testing of the resources, or in the design and reporting of this study.

## Reporting, dissemination and notification of results

We will provide all of the participating schools and school authorities with a report of the main findings of the study as soon as the analyses have been completed and independently checked by at least two referees who were not involved in the trial. We will invite the school authorities, head teachers, and participating primary-five teachers to meetings where we will present and discuss the findings of the study. We will offer the IHC primary school resources to schools in the control group following the trial, regardless of the findings.

We will actively disseminate the results of this trial through publications and presentations. All of the resources will be made available on the project website and Testing Treatments interactive. If the primary school resources are effective, we will actively disseminate them internationally through our international advisory group, the Cochrane Collaboration, the Evidence-informed Policy Network, the World Health Organisation, UNICEF, the Campbell Collaboration and other relevant networks and organisations. Publications and the resources will be open access, allowing free noncommercial use, distribution, reproduction and further development, provided that the source is properly cited.

## Discussion

So far as we are aware, this is the first randomised trial to evaluate the effects of an intervention to teach key concepts that are essential to improving people’s ability to critically assess claims made about the effects of treatments to primary school children [18–21]. It is unlikely that the IHC primary school resources alone will have a measurable effect on health outcomes. Primary school children do not make many decisions on their own, and there are many other factors that affect decision-making and health behaviours. Nonetheless, these skills are essential for informed participation in personal and societal health care decisions, as well as for coping with the flood of information making claims about treatment effects.

It is important to introduce these key concepts at a young age to lay a foundation for future learning. Whether what is learned is sustained or not, it would be desirable to reinforce what is learned and to introduce additional key concepts as part of a spiral curriculum [44]. Thus, it is important to evaluate whether the resources have an important effect on children’s ability to assess treatment claims, regardless of whether this has a measurable impact on health or how long what is learned is retained. If there is an important effect, the implication will be that consideration should be given to using these resources, or similar interventions, and to build upon this in the future. If there is not an important effect, consideration should be given to why they did not work and how to design a more effective intervention. We will conduct a process evaluation to explore why the resources did, or did not, have intended effects and explain variations in effects [45]. In the process evaluation, we will also explore other potential adverse and beneficial effects than those that were measured in this trial, ways in which use of the primary school resources could be scaled up (assuming they are effective), and the impact of the intervention on teachers and parents.

In summary, we believe that the findings of this trial will have important implications for children, their parents and teachers, head teachers, policy-makers, and anyone with an interest in health literacy or evidence-informed decision-making.

## Additional files

Additional file 1: The Claim Evaluation Tools. (PDF 997 kb)

Additional file 2: Setting a standard for the ‘Claim 12’ and ‘Claim 9’. (DOCX 25 kb)

Additional file 3: Data collection form for teachers. (DOCX 15 kb)

Additional file 4: SPIRIT 2013 Checklist: IHC School Trials’ Checklist. (DOC 120 kb)

Additional file 5: Safety Monitoring and Adverse Events Form. (DOCX 28 kb)

Additional file 6: School authority (headteacher) Informed Consent Form – English. (DOCX 29 kb)

Additional file 7: Research participant (teacher’s) Informed Consent Form – English. (DOCX 27 kb)

## Abbreviations

IHC: Informed Health Choices
UNESCO: United Nations Educational, Scientific and Cultural Organisation
UNICEF: United Nations Children’s Fund
