# Supplementary material for: Does the use of the Informed Healthcare Choices (IHC) primary school resources improve the ability of grade-5 children in Uganda to assess the trustworthiness of claims about the effects of treatments: protocol for a cluster-randomised trial
Source: Trials. 2017 May 18;18:223. doi: 10.1186/s13063-017-1958-8 (PMC5437593; doi:10.1186/s13063-017-1958-8)
Supplement: Supplementary file 3 — Data collection form for teachers. (DOCX 15 kb) [file 13063_2017_1958_MOESM3_ESM.docx]

The Informed Healthcare Choices Project

**Data Collection Form for Teachers**

Date: _________________________________________________________________________

| How old are you? (Age) |  |
| --- | --- |
| Gender? (Male/Female) |  |
| What is your level of Education? (Degree, diploma, Certificate or other) |  |
| How long have you worked as a primary school teacher? (No. of years in teaching profession) |  |
| What subjects do you teach at school? | 1. (Main Subject)  2.  3.  4.  5.  6. |
| How many classes (lessons) do you teach per week? |  |
| What is your average class size (how many children are in your class on an average day? |  |
| Do you teach at any  other school in addition to this one? |  |
