# Supplementary material for: Does the use of the Informed Healthcare Choices (IHC) primary school resources improve the ability of grade-5 children in Uganda to assess the trustworthiness of claims about the effects of treatments: protocol for a cluster-randomised trial
Source: Trials. 2017 May 18;18:223. doi: 10.1186/s13063-017-1958-8 (PMC5437593; doi:10.1186/s13063-017-1958-8)
Supplement: Supplementary file 5 — Safety Monitoring and Adverse Events Form. (DOCX 28 kb) [file 13063_2017_1958_MOESM5_ESM.docx]

# INFORMED HEALTHCARE CHOICES PROJECT

# Safety Monitoring and Adverse Events Form.

| **AE= Adverse events. SAE= Serious Adverse Events.**  This report must be used to notify the reviewing Makerere University School of Medicine Institutional Review Board (Mak CHS-IRB) of AE and SAE that occur on site during the research project. The Principal Investigator (PI) should be notified regarding any AE or SAE (within 24 hours). It will be the responsibility of the PI to notify the reviewing IRB, if the AE or SAE definitely or possibly has material impact. |
| --- |

1. **STUDY TITLE**: Evaluation of resources to teach children in low-income countries to assess claims about treatment effects.
2. **STUDY OVERVIEW:** This is a two-arm cluster randomised trial of primary schools in the central region of Uganda. The study will be taking place in over one hundred schools during the second term of the academic year 2016. Primary five children in the selected schools that fall in the intervention arm will participate in lessons over a period of approximately twelve weeks using Informed Healthcare Choices resources. Primary five children in the control arm will continue with the current curriculum as usual. At the end of the term, all the children in both arms (control and intervention) of the trial will complete the CLAIM evaluation tool, which consists of multiple-choice questions that assess an individual’s ability to apply concepts that people must be able to understand and apply to assess treatment claims and to make informed healthcare choices.
3. **CONFIDENTIALITY:** All materials collected are for research purposes only and data will be kept in strict confidence.
4. **ADVERSE EVENT INFORMATION**: Any untoward or unfavorable occurrence in a human study participant, including any abnormal sign (e.g. abnormal physical exam or laboratory finding), symptom, or disease, temporally associated with the participants’ involvement in the research is considered an adverse event.

A Serious Adverse Event (SAE) could result in

- Death
- Prolonged hospitalization
- Causes persistent or significant disability etc.

This study has been given a very low risk rating by the ethics review board. **We therefore anticipate that participation in this research study presents no risk to the individual that may result in an adverse event or a serious adverse event.**

1. **EXPECTED RISKS:** None in relation to participating individuals’ health.
2. **UNANTICIPATED PROBLEMS:** Unanticipated problems, usually not directly related to the study may affect the delivery of the intervention. Any arising events that may affect delivery of the intervention should be reported to the Principal Investigator in 48 hours.

Unanticipated problems could include:

- Student strikes
- Teacher strikes
- War
- Epidemic outbreaks (Ebola, Cholera etc)

**AE, SAE and UP EVENT REPORTING FORM**

| **Event ID** | **Date of Event** | **Relationship to the research protocol** | **Has the event been reported to the P.I?** *Please tick (√) if yes* | **Site of the Event.** (*Provide school name and indicate the District*) | **Is the event considered to have a material impact*?** | **Has the event been reported as required by the IRB?** *Please tick (√) if yes* | **Action taken/recommended. (***If any, please give details below)* |
| --- | --- | --- | --- | --- | --- | --- | --- |
| **001** |  | **Definitely related**    **Probably related.**    **Possibly related.**    **Unrelated.**    **Unknown** |  |  | **Definitely**    **Possibly**    **No** |  |  |

Principal Investigator’s Contact details.

**Ms. Allen Nsangi**

Makerere University College of Health Sciences.
New Mulago Hospital Complex, Administration Building, Second Floor.
P.O.Box 7072, Kampala Uganda
Mobile: 0773333629 Email: nsallen2000@yahoo.com
