# Supplementary material for: Does the use of the Informed Healthcare Choices (IHC) primary school resources improve the ability of grade-5 children in Uganda to assess the trustworthiness of claims about the effects of treatments: protocol for a cluster-randomised trial
Source: Trials. 2017 May 18;18:223. doi: 10.1186/s13063-017-1958-8 (PMC5437593; doi:10.1186/s13063-017-1958-8)
Supplement: Supplementary file 6 — School authority (headteacher) Informed Consent Form – English. (DOCX 29 kb) [file 13063_2017_1958_MOESM6_ESM.docx]

**SCHOOL AUTHORITY (HEADTEACHER) INFORMED CONSENT FORM-ENGLISH**

| **Project title:** | Informed Healthcare Choices Project. Version 1.0  Dated: (1^st^ February 2016). |
| --- | --- |
| **Study Principal Investigator:** | Ms. Allen Nsangi |
| **Co-investigator:** | Dr. Daniel Semakula |
| **Addresses:** | Makerere University, College of Health Sciences  New Mulago Hospital Complex, Clinical Research Building, 2nd Floor  P.O Box 7072, Kampala, Uganda  Phone: 0312109456 |
| **Date/Revision:** | Version 1.0 (1^st^ February 2016) |

1. **INTRODUCTION**

The Informed Healthcare Project is a research collaboration that seeks to improve health literacy by developing and testing of resources that teach primary school children to assess claims about benefits and harms of treatments.

In Uganda, the study is going to be conducted by researchers from Makerere University College of Health Sciences in several schools in the central region.

The information in this document is meant to help you decide whether or not your school should take part in this study but first there a few things to note.

- Your school has been randomly selected to participate in this research because it is categorised as either government or private and is located in one of the selected districts in the central region of Uganda.
- We anticipate that once you agree on behalf of your school to participate, the school will be in the study for a period of one school term (second term 2016), with follow-up after one year.
- Two copies of this consent form will be offered to your school for future reference.
- Please feel free to ask if you have any questions or concerns at any time before the start or during the conduct of the research.

1. **WHY IS THIS research BEING CONDUCTED?**

The ability to appraise claims about benefits and harms of treatment is crucial for informed health decision making. This research aims to enable children in low-income countries like Uganda to acquire and retain skills that will help them make informed healthcare choices by improving their ability to obtain, process and understand health information.

1. **HOW will The study be conducted?**

The study will take place in over one hundred schools in the central region of Uganda, during the second term of the academic year 2016, with some schools allocated to the intervention arm and others to the control arm. All the selected schools will be allocated to either the intervention arm or to the control arm using computer-generated randomization.

Primary five children in the intervention arm together with their teachers will participate in lessons over a period of approximately twelve weeks (one school term). Each child in primary five in the intervention arm of the study will get a textbook and an exercise book, and each participating primary five teacher will be given a teachers’ guide. There are nine lessons in the textbook and each lesson requires about 80 minutes of class time.

In the control arm, primary five children will continue with the curriculum as normal during the school term.

The CLAIM evaluation tool consists of multiple-choice questions that assess an individual’s ability to apply concepts that people must be able to understand and apply to assess treatment claims and to make informed healthcare choices. A version that covers only concepts covered by the textbook will be used to evaluate the primary school resources that we are testing.

All the children in both arms of the trial will complete the questionnaires in their classrooms at the end of the term. This requires approximately one hour. We will administer the questionnaires again after one year to find out if the children retained what they learned.

All the schools that were allocated to the control arm will also get the children’s book and teachers’ guides after the trial has been concluded and the CLAIM questionnaires have been collected.

1. **Possible benefits to your school**

Children in your school may gain new knowledge that will enable them to obtain, process and understand health information that they may need to make appropriate healthcare decisions. Your school can keep the textbooks that we are evaluating and we will invite the primary five teachers to a training workshop.

There will be no other direct benefit to your school from participating in this study and there is no promise of gaining any financial benefit from the project currently or in the future.

1. **Cost to the school**

The school will incur no cost other than the time taken to participate in the study.

1. **Compensation**

The school will not gain any form of compensation, monetary or otherwise for participating in the study, but appropriate daily expenses for lunch and transport will be reimbursed to any teachers that attend any study-related meetings or workshops.

1. **Confidentiality**

The information that we collect during the conduct of this research will be kept confidential in accordance with the ethical standards agreed upon by the local and international organizations governing the conduct of research involving human participants.

Any information resulting from this study, if published in scientific journals or presented at scientific meetings, will not reveal your school’s identity.

1. **Right to Refuse/Withdraw**

Participation in this research is purely voluntary and you are free to decline on behalf of your school to take part or withdraw at any time without any repercussions.

1. **Questions ABOUT THE RESEARCH**

In case of any further questions, please contact the Principal Investigator, Ms. Allen Nsangi, at Makerere University College of Health Sciences, P.O. Box 7072, Kampala Uganda: Tel: 0773333629 or email [nsallen2000@yahoo.com](mailto:nsallen2000@yahoo.com).

If you have questions regarding the ethics of this research, you may contact Prof. James Tumwine, Chairperson, Mak CHS School of Medicine Research and Ethics Committee. Tel: 0414 531875.

1. **DECLARATION OF Consent**

I have read the above information about this study and all my questions have been answered. I feel that I have been given enough information and time to consider my decision on behalf of my school to participate in this study. I fully understand that by signing this form, I do not waive any of my legal rights, nor does it relieve the study investigators of their liability. By signing this form, I confirm that I have been informed about the research study in which I am on behalf of my school voluntarily agreeing to take part.

Having understood all the information pertaining to this study, I therefore agree to my school’s participation in this study by appending my signature and name below.

| **ON BEHALF OF THE SCHOOL**  Name:  ____________________________________ | Signature:  _________________________________ |
| --- | --- |
| Date:  ___________________________________ | Tel number:  _________________________________________ |

| **SCHOOL DETAILS**  Name of the school:  ________________________________________ |  |
| --- | --- |
| District:  ________________________________________ |  |
