# Supplementary material for: Does the use of the Informed Healthcare Choices (IHC) primary school resources improve the ability of grade-5 children in Uganda to assess the trustworthiness of claims about the effects of treatments: protocol for a cluster-randomised trial
Source: Trials. 2017 May 18;18:223. doi: 10.1186/s13063-017-1958-8 (PMC5437593; doi:10.1186/s13063-017-1958-8)
Supplement: Supplementary file 7 — Research participant (teacher’s) Informed Consent Form – English. (DOCX 27 kb) [file 13063_2017_1958_MOESM7_ESM.docx]

**RESEARCH PARTICIPANT (TEACHER’S) INFORMED CONSENT FORM-ENGLISH**

| **Project title:** | Informed Healthcare Choices Project. Version 1.0  Dated: (1^st^ February 2016). |
| --- | --- |
| **Study Principal Investigator:** | Ms. Allen Nsangi |
| **Co-investigator:** | Dr. Daniel Semakula |
| **Addresses:** | Makerere University, College of Health Sciences  New Mulago Hospital Complex, Clinical Research Building, 2nd Floor  P.O Box 7072, Kampala, Uganda  Phone: 0312109456 |
| **Date/Revision:** | Version 1.0 1^st^ February 2016 |

1. **INTRODUCTION**

The Informed Healthcare Project is a research collaboration that seeks to improve health literacy by developing and testing of resources that teach primary school children to assess claims about benefits and harms of treatments.

In Uganda, the study is going to be conducted by researchers from Makerere University College of Health Sciences in several schools in the central region.

The information in this document is meant to help you decide whether or not to take part in this study but first there a few things to note.

- You are being asked to participate in this research because you are a teacher at one of the participating schools in Uganda and are of legal consenting age.
- We anticipate that once you agree to participate you will be in the study for a period of one school term (second term 2016), with follow-up after one year.
- You will be offered a copy of this form for your future reference.
- Please feel free to ask if you have any questions or concerns at any time before the start or during the conduct of the research.

1. **WHY IS THIS research BEING CONDUCTED?**

The ability to appraise claims about benefits and harms of treatment is crucial for informed health decision making. This research aims to enable children in low-income countries to acquire and retain skills that will help them make informed healthcare choices by improving their ability to obtain, process and understand health information.

1. **HOW will The study be conducted?**

The study will take place in over one hundred schools in the central region of Uganda, during the second term of the academic year. Primary five children in the selected schools together with their teachers will participate in lessons over one school term.

All the selected schools will be allocated to either the intervention or control arm using computer-generated randomization. A total of 55 schools will be in the control arm and another 55 schools in the intervention arm.

If your school is allocated to the intervention arm, you will be expected to teach nine lessons during the school term, using the Informed Healthcare Choices school resources. Each child in your primary five class will get a text book and an exercise book, and you will be given a teachers’ guide.

If your school is allocated to the control arm, your primary five class will continue with the curriculum as normal during the school term.

The CLAIM evaluation tool consists of multiple-choice questions that assess an individual’s ability to apply concepts that people must be able to understand and apply to assess treatment claims and to make informed healthcare choices. A version that covers only concepts covered by the textbook will be used to evaluate the primary school resources that we are testing.

All the children in both arms of the trial will complete the questionnaires in their classrooms at the end of the term. This requires approximately one hour. We will administer the questionnaires again after one year to find out if the children retained what they learned.

After the trial has been concluded and the CLAIM questionnaires have been collected, all schools that were allocated to the control arm will also get the children’s book and teachers’ guides.

1. **Possible risks to you**

We anticipate that your participation in the study/research presents no risk to you as an individual. However, participation in this study might in some way interfere with your work, as you will be required to participate in study activities during your usual work hours.

1. **Possible benefits to you**

Participation in this study may improve your own ability to obtain, process and understand health information that you need to make appropriate healthcare decisions, as well as your ability to teach these skills to your students. Your school can keep the textbooks that we are evaluating and we will invite participating teachers to a training workshop.

There will be no other direct benefit to you from participating in this study and there is no promise of gaining any material or financial benefit from the project currently or in the future.

1. **Cost to you**

You will incur no cost other than the time taken to participate in the study.

1. **Compensation**

You will not gain any form of compensation, monetary or otherwise for participating in the study, but appropriate daily expenses for lunch and transport will be reimbursed if you attend any study-related meetings or workshops.

1. **Confidentiality**

The information you give during the conduct of this research will be kept confidential in accordance with the ethical standards agreed upon by the local and international organizations governing the conduct of research involving human participants.

Any information resulting from this study, if published in scientific journals or presented at scientific meetings, will not reveal your identity.

1. **Right to Refuse/Withdraw**

Your participation in this research is purely voluntary and you are free to decline to take part or withdraw at any time without any repercussions.

1. **Questions ABOUT THE RESEARCH**

In case of any further questions, please contact the Principal Investigator, Ms. Allen Nsangi, at Makerere University College of Health Sciences, P.O. Box 7072, Kampala Uganda: Tel: 0773333629 or email nsallen2000@yahoo.com

If you have questions in regarding the ethics of this research, you may contact Prof. James Tumwine, Chairperson, Mak CHS School of Medicine Research and Ethics Committee. Tel: 0414 531875.

1. **DECLARATION OF Consent**

I have read the above information about this study and all my questions have been answered. I feel that I have been given enough information and time to consider my decision to participate in this study. I fully understand that by signing this form, I do not waive any of my legal rights, nor does it relieve the study investigators of their liability. By signing this form, I confirm that I have been informed about the research study in which I am voluntarily agreeing to take part.

Having understood all the information pertaining to this study I therefore agree to my participation in this study by appending my signature and name below.

| **Research Participant**  Name:  ____________________________________ | Signature:  _________________________________ |
| --- | --- |
| School:  ___________________________________ | Date and Tel number:  _________________________________________ |
